# Coordinated Activation of VEGF/VEGFR-2 and PPARδ Pathways by a Multi-Component Chinese Medicine DHI Accelerated Recovery from Peripheral Arterial Disease in Type 2 Diabetic Mice

**DOI:** 10.1371/journal.pone.0167305

**Published:** 2016-12-08

**Authors:** Shuang He, Tiechan Zhao, Hao Guo, Yanzhi Meng, Gangjian Qin, David A. Goukassian, Jihong Han, Xuimei Gao, Yan Zhu

**Affiliations:** 1 Tianjin State Key Laboratory of Modern Chinese Medicine, Tianjin University of Traditional Chinese Medicine, Tianjin, China; 2 Research and Development Center of TCM, Tianjin International Joint Academy of Biotechnology & Medicine, Tianjin, China; 3 Molecular Cardiology Research Institute, Tufts Medical Center and Tufts University School of Medicine, Boston, United States of America; 4 Department of Medicine-Cardiology and Department of Molecular Pharmacology and Biological Chemistry, Northwestern University Feinberg School of Medicine, Chicago, United States of America; 5 Center of Biomedical Research, Tufts University School of Medicine, Boston, United States of America; 6 State Key Laboratory of Medicinal Chemical Biology, and Collaborative Innovation Center for Biotherapy, Nankai University, Tianjin, China; Massachusetts General Hospital, UNITED STATES

## Abstract

Diabetic mellitus (DM) patients are at an increased risk of developing peripheral arterial disease (PAD). Danhong injection (DHI) is a Chinese patent medicine widely used for several cardiovascular indications but the mechanism of action is not well-understood. We investigated the therapeutic potential of DHI on experimental PAD in mice with chemically induced as well as genetic (KKAy) type 2 DM and the overlapping signaling pathways regulating both therapeutic angiogenesis and glucose homeostasis. Compared with normal genetic background wild type (WT) mice, both DM mice showed impaired perfusion recovery in hind-limb ischemia (HLI) model. DHI treatment significantly accelerated perfusion recovery, lowered blood glucose and improved glucose tolerance in both DM models. Bioluminescent imaging demonstrated a continuous ischemia-induced vascular endothelial growth factor receptor 2 (VEGFR-2) gene expressions with a peak time coincident with the maximal DHI stimulation. Flow cytometry analysis showed a DHI-mediated increase in endothelial progenitor cell (EPC) mobilization from bone marrow to circulating peripheral blood. DHI administration upregulated the expression of vascular endothelial growth factor A (VEGF-A) and VEGF receptor-2 (VEGFR-2) in ischemic muscle. A cross talk between ischemia-induced angiogenesis and glucose tolerance pathways was analyzed by Ingenuity Pathway Analysis (IPA) which suggested an interaction of VEGF-A/VEGFR-2 and peroxisome proliferator-activated receptor δ (PPARδ)/peroxisome proliferator-activated receptor γ (PPARγ) genes. We confirmed that upregulation of VEGF-A/VEGFR-2 by DHI promoted PPARδ gene expression in both type 2 diabetic mice. Our findings demonstrated that a multi-component Chinese medicine DHI effectively increased blood flow recovery after tissue ischemia in diabetic mice by promoting angiogenesis and improving glucose tolerance through a concomitant activation of VEGF-A/VEGFR-2 and PPARδ signaling pathways.

## Introduction

There is a high prevalence of peripheral artery disease (PAD) and the lower extremities are its most common sites. Individuals with PAD and diabetes mellitus (DM) co-morbidity have a seven-fold higher risk of critical limb ischemia and a five-fold higher risk of amputation compared with PAD patients without DM [1]. Since diabetic patients have a four times greater risk of developing PAD compared to the general population, it is accepted that there is a close relationship between hyperglycemia and vascular complications [2]. Therefore, diabetic patients have much worse lower-extremity function and a higher risk of amputation [3]. Ample evidence suggests that DM affects the function of blood vessel, which may lead to a greater severity of disease.

Novel pharmacological approaches, based mainly on the knowledge gained from studying therapeutic angiogenesis, have been developed and applied clinically for PAD. Angiogenesis is the growth of new vessels from pre-existing vascular structures. Vascular endothelial growth factors (VEGFs), well-known pro-angiogenic factors involved in blood vessel growth during development and post-natal angiogenesis [4], mediate their biological effects through binding to their receptors, VEGF receptors 1 and 2 (VEGFR-1 and VEGFR-2). The Ligand-receptor interactions of VEGF and VEGFR play a critical role in perfusion recovery following HLI. It has been shown that VEGFR-2 is a dominant receptor that mediates post-natal angiogenesis [1]. We hypothesized that the impaired perfusion recovery in HLI of Type 2 DM mice may be associated with decreased expression of VEGF-A and VEGFR-2. Peroxisome proliferator-activated receptor δ (PPARδ) is a ligand-activated transcription factor that belongs to the nuclear receptor super-family, which also includes PPARα and PPARγ. Among these three isotypes, PPARδ is the most important regulator for executing key cellular functions in the heart, liver, colon, and skeletal muscle. Both *in vitro* and *in vivo* studies have shown that PPARδ is pro-angiogenic and plays an important role in the activation of angiogenic pathways [5].

On the other hand, numerous studies have shown that peripheral blood (PB) or bone marrow (BM)-derived EPCs are mobilized to ischemic tissue and contribute significantly to angiogenesis, collateral vessel development and augment blood flow recovery in ischemic damaged tissues in HLI model. Animal studies suggest that transplanted BM cells or BM-derived EPCs contribute to the development of collateral vessels. Blood flow recovery and capillary density in the ischemic hind-limb were markedly improved, and the rate of limb loss was significantly reduced. Therefore pharmacologic and/or biological agents that could mobilize EPCs into peripheral blood and improve recruitment and incorporation of EPCs to the ischemic tissue would enhance angiogenesis and improve the perfusion recovery [6,7].

Danhong injection (DHI) is a patent injection medicine made from the extracts of Radix Salviae Miltiorrhizae and Flos Carthami, which are two TCMs with a property of activating blood circulation and removing blood stasis. DHI is widely prescribed for the treatment of cardiovascular and cerebrovascular disease in clinical practice. We have previously identified 11 polyphenolic acids in DHI using ultra-performance liquid chromatography (UPLC) coupled with UV detection [8]. With a newly developed proton nuclear magnetic resonance (1H NMR) profiling method, we also simultaneously identified and quantified 23 primary metabolites together with 7 polyphenolic acids in DHI [9]. Recently, other investigators have further characterized and identified a total of 63 compounds, including 33 phenolic acids, 2 C-glycosyl quinochalcones, 6 flavonoid O-glycosides, 4 iridoid glycosides, 6 organic acids, 5 amino acids, and 3 nucleosides in DHI [10]. Our previous studies revealed that DHI increased endothelial-dependent vasorelaxation *in vivo* and *ex vivo* in rat aortas via prostacyclin/ cyclooxygenase-2 pathway [11]. DHI could prevent ischemia/reperfusion-induced brain damage through activating Nrf2/ARE signaling pathway [12]. Interestingly, DHI protected rat cardiac myocyte damage induced by overdose arginine vasopressin (AVP) and significantly decreased the injury of both primary rat neuronal cells and rat cardiac myocytes. The ability of DHI to reinstate AVP level may be one of the mechanisms of its brain and heart co-protection effects [13]. DHI also played an important role in suppressing inflammatory responses through inhibiting the NF-κB signaling pathway [14]. It may exert anti-cardiac hypertrophic effects by regulating p38 and NF-κB pathway [15]. Danshensu (DSS) was identified as a major vasorelaxation factor in DHI [11]. DSS could also improve circulation in smaller arteries, reduce ROS generation [16,17], inhibit cardio fibroblast proliferation and improve collagen synthesis [18,19], inhibit cell apoptosis [20] and protect heart against ischemia reperfusion injury [21,22]. A previous study showed that phenolic acids were the potential components responsible for the antioxidant activity of DHI [8]. Researches have shown that Hydroxysafflor yellow A (HSYA) may be a major active component of DHI from Flos Carthami. It exerts a variety of effects upon the cardiovascular system. For example, HSYA inhibits thrombosis and platelet aggregation, improves congestive cardiac failure in rats by suppressing ET-1 and iNOS and reduces oxidative stress in infarcted tissue [23]. HSYA has also been shown to reduce phenylephrine or KCl-induced vasoconstriction and to reduce blood pressure and heart rate [24].

Mouse model of hind-limb ischemia, with surgical ligation and excision of the femoral artery, has been used extensively to study processes that are involved in ischemia-induced perfusion recovery following vessel occlusion. Using this model, several studies have shown that perfusion recovery is impaired in diabetic mice [25]. KKAy mouse is a transgenic model of spontaneous development of type-2 diabetes (T2D). This congenic strain is established by transfer of the yellow obese gene (Ay) into KK mice with moderate hyperglycemia through repeated crossing of yellow obese mice [26]. This mouse is widely used as an experimental model for T2D. VEGFR-2-luc transgenic mice carry a transgene containing a 4.5-kb murine VEGFR-2 promoter and a modified firefly luciferase cDNA. The VEGFR-2 promoter drives the expression of luciferase to allow monitoring of the VEGFR-2 expression by bioluminescence imaging (BLI) [27]. Type 2 diabetes were induced by a high-fat diet (HFD) combined with low-dose streptozotocin (STZ) treatment [28]. In this model, after induction of hind-limb ischemia (HLI) therapeutic angiogenesis and VEGFR-2 expression can be visualized and monitored *in vivo* noninvasively and recovery of the ischemic limb measured continuously [27].

The goal of this study is to explore the effects of DHI on PAD with diabetes mellitus co-morbidity. Using the two independent mouse models of diabetic PAD described above, we demonstrated that DHI effectively increased blood flow recovery after tissue ischemia in diabetic mice by promoting angiogenesis and improving glucose tolerance through a concomitant activation of VEGF-A/VEGFR-2 and PPARδ signaling pathways.

## Materials and Methods

### Reagents

Danhong injection (DHI, Batch No.12081024077, 10mL/ampulla) comprising 750g Salvia miltiorrhiza, 250g Safflower and 7g Sodium chloride, was supplied by Heze Buchang Pharmaceutical Co., Ltd, Shandong, China.

### Animals

Female 6-week-old KK/Upj-Ay/J and 6-week-old C57BL/6J mice were purchased from HFK Bioscience Co, Ltd, (Beijing, China). Homozygous VEGFR-2-luc males were obtained from 3 transgenic breeding colonies currently maintained in a barrier room of Tianjin International Joint Academy of Biotechnology and Medicine (TJAB). These mice were housed in a 12-hour light/dark cycled facility with free access to food and water. All experiments were reviewed and approved by the Committee of Ethics on Animal Experiments at the TJAB (TJAB-JY-2011-002) and were carried out under the Guidelines for Animal Experiments at the Tianjin University of Traditional Chinese Medicine. Chloral hydrate (4%) was used as anesthetizing agent to achieve quick sedation in terminal tissue harvesting and blood drawing experiments. For invasive procedures, mice were anesthetized by inhalation of 3% isoflurane driven by 100% O_2_ for induction, then maintained at 2% isoflurane (100 ml/min O_2_). Animals were euthanized by cervical dislocation. All precautions were taken to minimize suffering.

### Experimental design

Since DHI is administered intravenously at a dosage of 10 ml per day in clinics, we converted to a concentration of 1.3 ml/kg for mouse by intraperitoneal injection (i.p.) once daily. After operative excision of one femoral artery, KKAy mice were randomly divided into four groups (Table 1) and daily i.p. injected with saline (1.3 ml/kg body weight, vehicle group, n = 7), DHI (1.3 ml/kg body weight, Danhong group, n = 7), rosiglitazone (1mg/kg body weight, rosiglitazone group, n = 3), and AMD3100 (5mg/kg body weight, AMD3100 group, n = 3) respectively for 35 days. C57BL/6J mice daily injected with saline (1.3 ml/kg body weight, C57 group, n = 7) were used as normal controls. Homozygous VEGFR-2-luc males were divided into four groups (Table 2). Except for three non-diabetic mice, other homozygous VEGFR-2-luc males were induced by high fat diet for one month, then received daily i.p. injections of 50mg/kg STZ freshly dissolved in 10 mmol/L citrate buffer (pH 4.5), for 5 consecutive days to induce type 2 diabetic models. After operative excision of one femoral artery, diabetic mice were randomly divided into three groups and daily i.p. injected with saline (1.3 ml/kg body weight, vehicle group, n = 7), DHI (1.3 ml/kg body weight, Danhong group, n = 7), metformin (129 mg/kg body weight, metformin group, n = 3) respectively. DHI and metformin were injected i.p. once daily for 35 days. Non-diabetic mice were used as normal group and daily i.p. injected with saline (1.3 ml/kg body weight, non DM group, n = 3).

**Table 1:** Groups of KKAy mice and WT mice.

Group	Number	Dosage per day	Duration (days)
C57	7	1.3 ml/kg	35
KKAy+vehicle	7	1.3 ml/kg	35
KKAy+rosiglitazone	3	1 mg/kg	35
KKAy+AMD3100	3	5 mg/kg	35
KKAy+DHI	7	1.3 ml/kg	35

**Table 2:** Groups of VEGFR-2-luc mice.

Group	Number	Dosage per day	Duration (days)
Non DM	3	1.3 ml/kg	35
DM+vehicle	7	1.3 ml/kg	35
DM+metformin	3	129 mg/kg	35
DM+DHI	7	1.3 ml/kg	35

### Blood glucose measurement and, glucose tolerance test (GTT)

Blood samples were collected from lateral tail veins. Blood glucose was measured with an automatic glucometer (Roche ACCU-CHEK Active, Germany) at 2 p.m. every 10 days. Fasted mice were i.p. injected with dextrose (2 g/kg). Glucose level was measured in tail blood before and at 15, 30, 60, and 120 min after dextrose injection using a glucometer.

### Murine hind-limb ischemic model

Mice were anesthetized with isoflurane and unilateral hind-limb ischemia was induced, as described [29]. The entire right hind limb femoral artery and vein were exposed and isolated from the inguinal region to the bifurcation of the saphenous/popliteal artery. Exposed vessels were ligated at their proximal and distal ends, and both vessels were excised. The intact perfused contra-lateral limb of each mouse was used as an internal control. After hind-limb ischemia, saline or drugs were administrated daily for 35 days. Mice were euthanized at the end of the study and muscle tissues were collected and either fixed in 4% paraformaldehyde or snap-frozen in liquid nitrogen for the future use.

### Laser Doppler perfusion imaging (LDPI)

Mice were anesthetized as above and hair was removed using an electric clippers. A serial, non-invasive assessment of ischemic limb micro-vascular perfusion was performed using the LDPI system (MoorLDLS, UK). Recovery of hind limb perfusion was monitored periodically and expressed as the ratio of perfusion in the ischemic limb to perfusion in the healthy limb as described before [30].

### Bioluminescent imaging *in vivo*

Bioluminescence imaging (BLI) was performed using a highly sensitive, cooled charge-coupled device camera mounted in a light-tight specimen box (IVIS® Lumina K Series III, PerkinElmer), with protocols similar to those described previously [31]. For imaging *in vivo*, mice were anesthetized with isoflurane and i.p. injected with D-luciferin (PerkinElmer) at 150 mg/kg body weight per manufacturer recommendations. Optical signal intensity of the VEGFR-2-luc mouse was acquired 5 min after D-luciferin administration. Regions of interest (ROI) from displayed images were identified on the ischemic sites and quantified as photons per second (p/s) using Living Image® software.

### Flow cytometry

To investigate the effects of DHI on EPC mobilization to circulating peripheral blood (PB) in response to tissue ischemia, the fluorescence-activated cell sorting (FACS) Caliber flow cytometer (Becton Dickinson, San Jose, CA, USA) was used to assess EPC mobilization. 100 μL of PB was collected from the inner canthus of KKAy mice. PB was incubated with Fluorescein isothiocyanate (FITC) anti-mouse Sca-1 (eBioscience, San Diego, CA, USA), and phycoerythrin (PE) anti-mouse Flk-1 (VEGFR-2, eBioscience) antibodies [32]. Isotype-identical antibodies (IgGs) were used as negative controls (eBioscience). After 30 min incubation, cells were lysed with red blood cell lysis buffer, washed with phosphate-buffered saline (PBS), and fixed in 1% paraformaldehyde before analysis. Each analysis included 100,000 events. Double positive Sca-1/Flk-1 cell from the mononuclear fraction were considered to be circulating PB EPCs [33].

### Capillary count

Muscles were processed for histology. Sections were stained with hematoxylin and eosin (H&E) for histopathologic evaluation. Sections were prepared from KKAy mice and stained for Tie-2 to confirm the endothelial phenotype. Sections were prepared from VEGFR-2-luc mice and stained for VEGFR-2 to confirm the endothelial phenotype. Capillaries were counted by a single observer blindfolded for the treatment conditions under a fluorescence microscope. Different fields was selected randomly from each group.

### Quantitative real-time reverse transcription–polymerase chain reaction analysis

Total RNA samples from the muscle tissues were isolated using *EasyPure*® RNA Kit (TRANSGEN BIOTECH) according to manufacturer’s protocols. RNA samples were subsequently reverse-transcribed to complementary DNA (cDNA) using Transcriptor First Strand cDNA Synthesis Kit (Roche) to obtain cDNA. The resulting cDNA was used as a template for real-time polymerase chain reaction (PCR) amplification using the following oligonucleotide primers:

5′- TGGTGAAGCAGGCATCTGAG-3′ (forward) and

5′- TGCTGTTGAAGTCGCAGGAG-3′ (reverse) primers for mouse GAPDH,

5′- TGTACCTCCACCATGCCAAGT -3′ (forward) and

5′- TGGAAGATGTCCACCAGGGT-3′ (reverse) primers for mouse VEGF-A, 5′-ACTGCAGTGATTGCCATGTTCT-3′ (forward) and

5′-TCATTGGCCCGCTTAACG-3′ (reverse) primers for mouse VEGFR-2,

5′-TCCATCGTCAACAAAGACGGG-3′ (forward) and

5′-ACTTGGGCTCAATGATGTCAC-3′ (reverse) primers for mouse PPARδ,

5′-AGGCCGAGAAGGAGAAGCTGTTG-3′ (forward) and

5′-TGGCCACCTCTTTGCTCTGCTC-3′ (reverse) primers for mouse PPARγ,

5′-CACAGAACCAGTTTCCATCATCCAGT-3′ (forward) and

5′-CATGTTCAGAGGGTTAGGGAGAGCA-3′ (reverse) primers for mouse CREBBP,

5′- CGGAAATCATATCCAACCAG-3′ (forward) and

5′- TGAGGACCGCTAGCAAGTTTG-3′ (reverse) primers for mouse PPARGC1A

The FastStart Universal SYBR Green Master (ROX) (Roche) was used in qRT-PCR to quantify the level of VEGF-A and VEGFR-2 with GAPDH as an internal control. The PCR reactions were setup in duplicates in 25 μL of total volumes including 2.5 μL of each primer, 12.5 μL of FastStart Universal SYBR Green Master (ROX), and 1 μL of template. Amplification was performed on C1000TMThermal Cycler (BIO-RAD, USA) for 45 cycles. The amplification and analysis were performed using C1000TMThermal Cycler sequence Detection System. Samples were compared using the relative C_T_ method. The fold increase or decrease was determined relative to a blank control after normalizing to a housekeeping gene using 2^-△△CT^, GAPDH.

### Ingenuity Pathway Analysis

Functional interactions between the angiogenesis and glucose tolerance were analysed by Ingenuity Pathway Analysis (IPA) tool (Ingenuity Systems, Redwood City, CA). Since our experimental results revealed DHI-mediated increase in the expression of VEGF-A and Flk-1 in both genetic and chemically induced diabetic mice in HLI models, we used the Map function of IPA to predict the potential up- and/or down-regulation of genes in the glucose tolerance pathway.

### Statistical analysis

Data analyses were performed by Origin 8.5.1 software (OriginLab, USA) and the significant difference was analyzed by SPSS 11.5 software (IBM, USA). The results were shown as mean ± standard error of mean from “n” number of experiments. Statistical significance was assessed by unpaired Student’s t test for observation between 2 groups or by analysis of variance test for comparison between multiple groups. A P value of <0.05 was considered to be statistically significant.

## Results

### DHI improved glucose homeostasis

**Fig 1:**
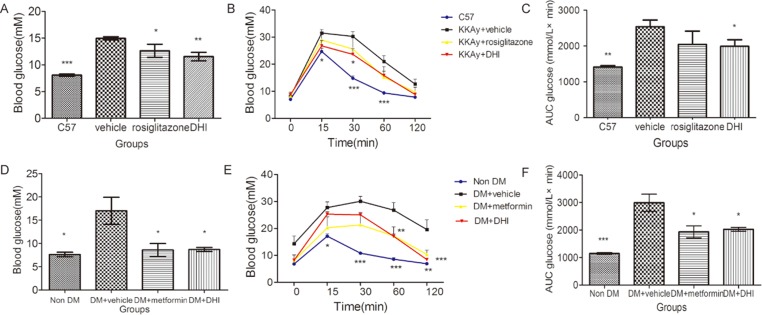
DHI improved glucose homeostasis. (A) In KKAy mice, the levels of blood glucose in DHI-treated and rosiglitazone-treated groups were significantly lower than that in vehicle-treated group. (B) Intraperitoneal glucose tolerance testing was performed in KKAy mice at the end of the feeding course, and the areas under the glucose tolerance tests curves were shown in (C). (D) In STZ-induced diabetic mice, the level of blood glucose in DHI-treated and metformin-treated groups were significantly lower than that in the vehicle-treated group. (E) Intraperitoneal glucose tolerance testing was performed in STZ-induced diabetic mice at the end of the feeding course, and areas under the glucose tolerance tests curves were shown in (F). Values are mean ± SEM. *P<0 .*05*, **P<0 .*01*, ***P<0 .*001 vs vehicle*.

After one-month treatment, the level of blood glucose in DHI-treated group was significantly lower than that in vehicle-treated group in both genetic and chemically-induced diabetic mouse models (Fig 1A and 1D). We used glucose tolerance test (GTT), a commonly used test to diagnose diabetes mellitus, to assess the blood glucose-regulating effects of DHI. As shown in Fig 1B and 1E, the blood glucose levels in all mice elevated sharply from 0 to 15 min after glucose loading and then gradually decreased over 120 min. Compared to control non-DM mice, there were noticeable and sustained decreases (p<0.05) in blood glucose levels at 30 and 60 min in DHI-treated mice, strongly suggesting that administration of DHI improved glucose homeostasis that was impaired in type 2 diabetic mice. Area under the curve (AUC), which represents the variation in glucose concentration from baseline over the test duration, was significantly smaller in DHI-treated than vehicle group mice (Fig 1C and 1F).

### DHI improved recovery of ischemic limb perfusion in both KKAy mice and STZ-induced diabetic mice

**Fig 2:**
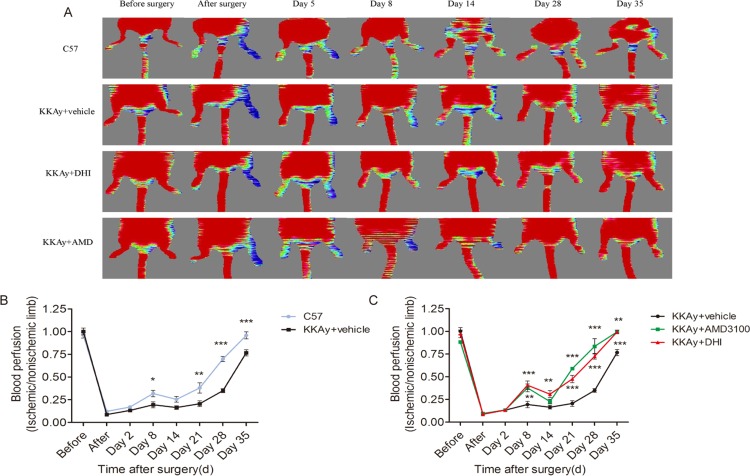
DHI improved perfusion of ischemic limbs in KKAy mice. (A) Representative images of laser Doppler perfusion analysis for WT control mice, KKAy mice treated with AMD3100, KKAy mice treated with or without DHI before surgery and at different time points after surgery. Low perfusion signals (dark blue) were observed in the ischemic hind limb, whereas high perfusion signals (red) were detected in KKAy mice treated with DHI on postoperative day 8 through 35 and in KKAy mice treated with AMD3100 on postoperative days 8, 21, 28, and 35. (B) Hind-limb perfusion recovery was impaired in untreated KKAy mice. The mean hind-limb blood flow was calculated as the ratio of ischemic (left) side to non-ischemic (right) side. (C) DHI or AMD3100 significantly improved perfusion recovery after HLI surgery. *P<0 .*05*, **P<0 .*01*, ***P<0 .*001 vs*. *KKAy+vehicle*.

**Fig 3:**
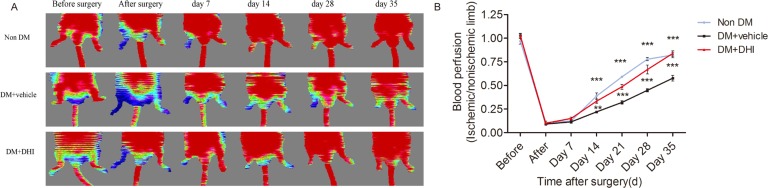
DHI improved perfusion of ischemic limbs in STZ-induced diabetic mice. (A) Representative images of laser Doppler perfusion analysis for non-diabetic mice, STZ-induced diabetic mice treated with or without DHI before surgery and at different time points after surgery. Low perfusion signals (dark blue) were observed in the ischemic hind limb, whereas high perfusion signals (red) were detected in diabetic mice treated with DHI on postoperative days 14, 21, 28 and 35. (B) Perfusion recovery was impaired in untreated STZ-induced diabetic mice. The mean hind-limb blood flow was calculated as the ratio of ischemic (right) side to non-ischemic (left) side. DHI showed a significantly improved perfusion recovery after HLI surgery. **P<0.*01*, ***P<0 .*001 vs*. *DM+vehicle*.

To evaluate the effect of DHI on blood perfusion in diabetic mice, laser Doppler perfusion imaging (LDPI) was performed before, immediately after and over 35 days after hind-limb ischemia surgery. Pre-operatively, the ratio of blood flow between the two hind limbs in all animals was set at 1.0. Immediately after induction of hind-limb ischemia, the ratio of the blood flow between the ischemic and contralateral normal non-ischemic limbs was 0.097±0.004 (Figs 2C and 3B). In diabetic mice injected with saline, blood flow recovered to a ratio of 0.67±0.09 after 35 days (Figs 2C and 3B), whereas in DHI- or AMD3100- treated diabetic mice and non-diabetic mice, the LDPI ratio was accelerated to 0.92±0.08, 0.99±0.04 and 0.89±0.07 after 35 days (Figs 2C and 3B). In addition, as early as 14, 21 to 28 days, DHI- or AMD3100-treated diabetic mice and non-diabetic mice showed a significantly better recovery of the limb perfusion (Figs 2B, 2C and 3B). Thus, blood-flow recovery was severely impaired in both genetic and induced diabetic mice and this deficiency was significantly improved by DHI or AMD3100 treatment.

### DHI promoted EPC mobilization to peripheral blood

**Fig 4:**
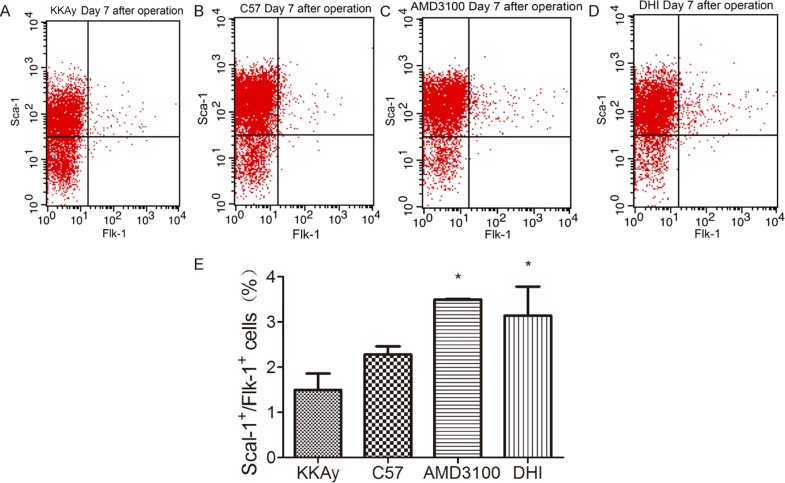
DHI increased EPC mobilization in KKAy mice. EPC (defined as Sca-1^+^/Flk-1^+^cells) mobilization after tissue ischemia was determined by flow cytometry in C57BL/6J mice and KKAy mice after administration of saline, DHI or AMD3100. DHI and positive-control AMD3100 both showed a significantly improved EPC mobilization after HLI surgery. *P<0 .*05 vs*. *KKAy+vehicle*.

To investigate the effect of DHI on EPC mobilization in response to tissue ischemia, the number of double positive Sca-1^+^/Flk-1^+^ cells in mononuclear fraction of peripheral blood were determined by flow cytometry in KKAy mice. EPC mobilization was enhanced by tissue ischemia. Administration of AMD3100 or DHI augmented substantially EPC mobilization after tissue ischemia in KKAy mice (Fig 4).

### DHI enhanced angiogenesis *in vivo* in VEGFR-2-luc diabetic mice post HLI

**Fig 5:**
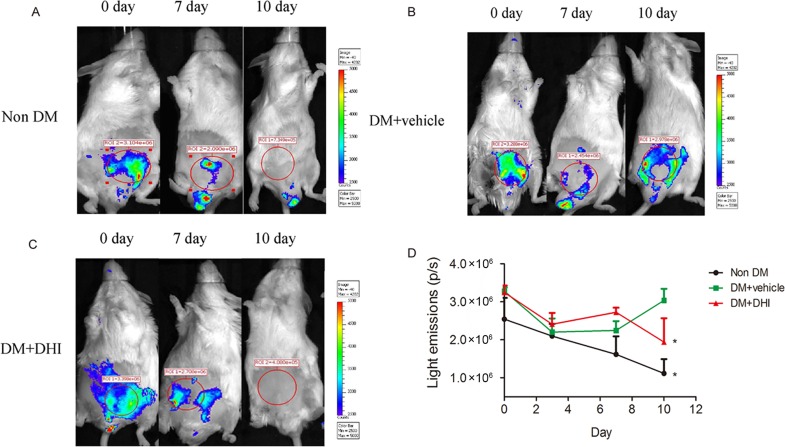
*In vivo* expression of VEGFR-2 in diabetic VEGFR-2-luc mice. (A-C) Representative Bioluminescent images of HLI mice were obtained at 0, 7 and 10 days under the same imaging conditions. (D) The dynamic measurement of bioluminescent intensities in non-DM, DM+vehicle and DM+DHI groups. Regions of interest (ROI) from displayed images were identified on the HLI sites and quantified as photons per second (p/s). Data is shown as mean ± SEM. n = 5 in each group. *P<0.*05*, *vs*. DM+vehicle.

In order to evaluate the effect of DHI on ischemia-induced angiogenesis *in vivo*, we chemically induced diabetes in VEGFR-2-luc mice, in which the expression of luciferase is driven by VEGFR-2 promoter to allow a direct visualization of angiogenesis using bioluminescent imaging. There was no difference in average bioluminescent intensities between control and treated groups before and 3 days after HLI surgery. However, compared to non-DM and DM+vehicle groups, HLI areas in DHI treatment group showed a higher bioluminescent intensities after 7 days, suggesting an upregulation of VEGFR-2 in the ischemic area (Fig 5A, 5B and 5C, middle) and a beginning of accelerate blood-flow perfusion recovery. Remarkably, 10 days after the treatment, bioluminescent intensities in DHI-treated group were not detectable while it was significantly increased in the DM+vehicle group (Fig 5B vs. 5C, far right; and Fig 5D). Undetectable expression of VEGFR-2 in DHI-treated mice by day 10 is an indication of decreased angiogenic signals in the ischemic limb, hence, suggesting a significantly improved angiogenesis in DHI-treated vs. vehicle-treated group *in vivo*.

### DHI reduced the incidence of limb necrosis

**Fig 6:**
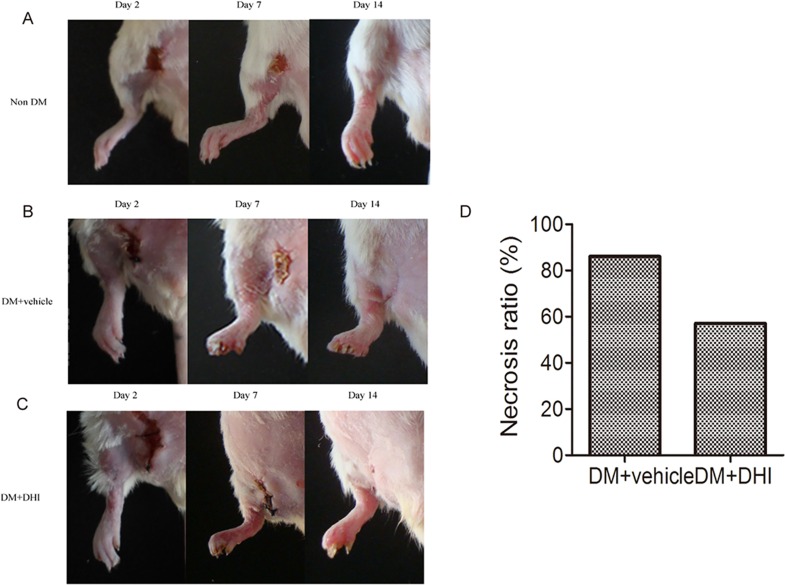
DHI decreased incidence of limb necrosis in diabetic mice post HLI. Representative images of hind limbs showing evidence of tissue necrosis. In DM+vehicle group, six out of seven ischemic limbs showed necrosis (86%), while in DM+DHI group, four out of seven ischemic limbs showed necrosis (57%).

In ischemic conditions, a significantly higher occurrence of limb necrosis was observed in STZ- induced diabetic mice compared to non-diabetic mice. Both the saline- and DHI-treated groups demonstrated significant toe necrosis by the second day after ischemia induction, whereas non-diabetic mice did not (Fig 6A, 6B and 6C, left). Compared to DM+vehicle group, there was approximately 30% decrease in mice with limb necrosis in DM+DHI group 14 days after HLI (Fig 6A, 6B and 6C, far right, and Fig 6D).

### DHI increased the capillary density in ischemic limb

**Fig 7:**
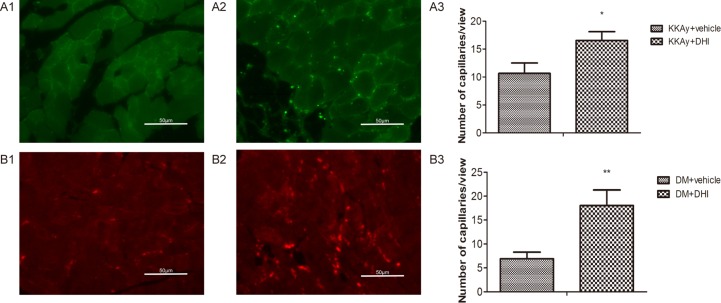
DHI increased capillary density in diabetic mice post HLI. Representative sections of ischemic limb muscle 5 weeks after DHI treatment. Capillaries were identified by Tie-2 or VEGFR-2 antibody staining. Ischemic limbs of KKAy mice injected with saline (A1) or DHI (A2) were shown where capillaries were identified by an anti-Tie-2 antibody. Ischemic limbs of STZ-induced diabetic mice injected with saline (B1) or DHI (B2) were shown where capillaries were identified by an anti- VEGFR-2 antibody. Quantitative analysis of capillary density of the ischemic region was performed at the end of week 5 for KKAy (A3) and STZ-induced (B3) diabetic mice. *P<0 .*05*, **P<0 .*01*.

Immunohistochemical staining for Tie-2 or VEGFR-2 was used to quantify the capillary density. In both genetic and STZ-induced diabetic mice, there was significant increase in the capillary density in the ischemic muscle tissue in DHI-treated mice, as compared with the vehicle-treated controls (Fig 7A and 7B). These results provided evidences that DHI-treatment protected muscle tissue from ischemia-induced necrosis by increasing capillary density.

### DHI increased the expression of VEGF-A and Flk-1 in both genetic and chemically induced diabetic mouse HLI models

**Fig 8:**
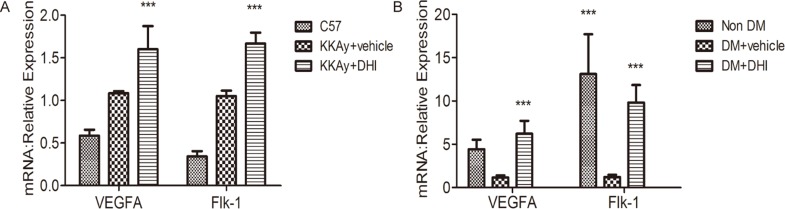
Increase of angiogenic factors in DHI-treated ischemic muscle. (A) Quantitative PCR showed DHI increased the expression of VEGF-A and Flk-1 in KKAy mice. (B) Quantitative PCR showed DHI increased the expression of VEGF-A and Flk-1 in STZ-induced diabetic mice. Data represent the means ± SEM, C57BL/6J: n = 5, KKAy+vehicle: n = 4, KKAy+DHI: n = 6; Non-DM: n = 3, DM+vehicle: n = 5, DM+DHI: n = 5; *vs*. *vehicle*, ***P<0 .*001*.

Quantitative PCR (qPCR) analyses were carried out to access whether DHI could influence the expression of angiogenesis-related genes in diabetic HLI mice. VEGF-A is a well-known angiogenic growth factor that induces formation of new capillaries in the ischemic tissue, whereas Flk-1 (VEGFR-2) is a predominant receptor of VEGF-A. As shown in Fig 8, the mRNA levels of VEGF-A and Flk-1 in DHI-treated group were significantly upregulated in both genetic and chemically induced diabetic mice compared with the control mice.

### DHI improved glucose tolerance by increasing the expression of PPARδ

**Fig 9:**
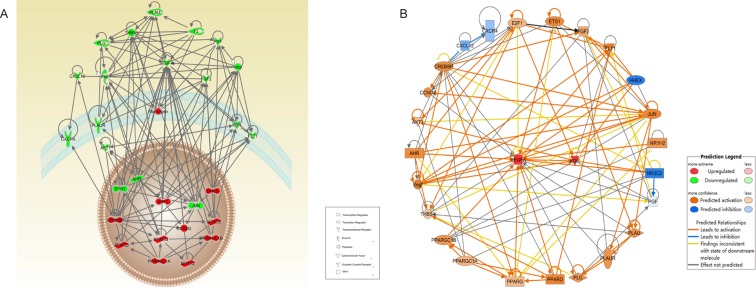
IPA indicated the overlap between angiogenesis and glucose tolerance networks. (A) Red colored genes are related to glucose tolerance and green colored genes are related to angiogenesis. The arrows indicate effects of regulated genes on other genes. (B) Orange colored are predicted to be activation and blue colored are predicted to be inhibited.

**Fig 10:**
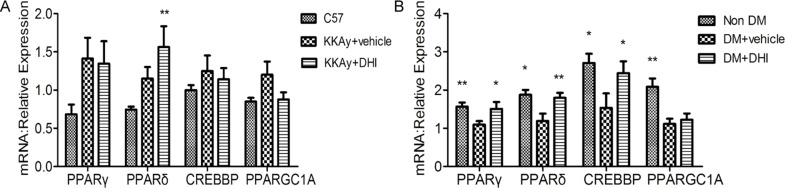
DHI increased PPARδ expression in ischemic muscle tissue. (A) Quantitative PCR showing increased PPARδ expression in genetic diabetic mice treated with DHI. (B) Quantitative PCR showing increased expression of PPARδ in chemically induced diabetic mice treated with DHI. Data represent the mean ± SEM, C57BL/6J: n = 5, KKAy+vehicle: n = 4, KKAy+DHI: n = 6; Non-DM: n = 3, DM+vehicle: n = 5, DM+ DHI: n = 5; *vs*. *vehicle*, **P<0 .*01*, *P<0.*05*.

Using Ingenuity Pathway Analysis (IPA), we identified interactions between angiogenesis and glucose tolerance pathways. As shown in Fig 9A, Red-colored genes were related to glucose tolerance and green-colored genes were related to angiogenesis. Continuous lines showed a direct interaction and arrows indicated regulated genes in the network. Since DHI increased the expression of VEGF-A and Flk-1 in both genetic and chemically induced diabetic mice post HLI, the Map function of IPA was used to predict the potential up- or down-regulated genes in the glucose tolerance pathway. As shown in Fig 9B, the function network analysis suggested that PPARγ, PPARδ, CREBBP and PPARGC1A are among the activated genes. A qPCR analysis was performed to validate this prediction. As shown in Fig 10A and 10B, DHI increased expression of PPARδ in both genetic and chemically induced diabetic mice, while in chemically induced diabetic mice, it also significantly increased the expression of PPARγ and CREBBP (Fig 10B). These results confirm the predicted activation of these genes by IPA analysis and indicated that DHI’s effects on improved glucose homeostasis is at least in part attributable to its upregulation of PPARδgene expression.

## Discussion

Peripheral arterial disease (PAD) is an atherosclerotic occlusive illness of the lower extremities. Diabetes mellitus (DM) is a well-recognized risk factor for microvascular disease that can lead to retinopathy and peripheral neuropathy [34]. PAD is a common vascular complication in patients with DM, most likely due to distal nature of microvascular pathology and its association with peripheral neuropathy. In a recent study, increased glycosylated hemoglobin (HbA1c) levels is shown to be associated with increased complications and prevalence of PAD in DM patients [35]. In addition to DM, the risk of developing PAD is increased in people with metabolic disease and obesity, and these risk factors also correlate with decreased ability to provide alternate conduits for impaired blood flow in the extremities [36]. Diabetes affects nearly every vessel in body, however, the persistent abnormal metabolic state associated with DM results in unique changes in arterial structure and function in the peripheral vasculature. Development of new non-invasive approaches, such as therapeutic angiogenesis, directed at revascularization and restoration of arterial structure and function are vitally necessary.

The VEGF/VEGFR ligand-receptor interactions play critical role in post-ischemic recovery following HLI. Studies have shown that in mature vessels, autocrine expression of low levels of VEGF is essential for vessel homeostasis whereas high levels of VEGF induce branching angiogenesis [37]. Numerous studies that evaluated the efficacy of proangiogenic gene therapy suggested that VEGF is a critical factor in therapeutic angiogenesis [38–40]. DHI is widely used in the treatment of cardiovascular diseases such as acute myocardial infarction, coronary heart and so on [41,42] and the beneficial effects could be seen in experimental ischemia models [43]. We hypothesized that the angiogenic properties of DHI might depend on VEGF regulation. Our results revealed that treatment with DHI upregulated levels of VEGF-A and VEGFR-2, a pathway that is crucial for the angiogenic processes. DHI-mediated increases in VEGF-A and VEGFR-2 were associated with improved tissue perfusion as early as one week after HLI and increased number of capillaries in the ischemic hind-limb. These findings suggest that exogenous DHI administration may enhance ischemia-induced angiogenesis in diabetic mice through VEGF-dependent mechanism. Of note, Liu et al. have reported that DHI improved renal functions by inhibiting expression of VEGF-A in kidneys [44]. In contrast to findings in kidney or results showed that DHI increased VEGF-A and VEGFR-2 expression in the ischemic muscle and led to accelerated blood flow restoration and improved post-ischemic revascularization. Thus, the comparative results of these two studies demonstrated the divergent effect of DHI on angiogenesis in the ischemia tissue versus kidneys. Although hypothetical, these divergent findings in two organ-tissues with and without tissue ischemia suggest that DHI treatment could regulate same angiogenic pathway (VEGF/VEGFR-2) in opposite directions. Even though, underlying molecular mechanisms of these findings are not known, we postulate that DHI-induced tissue responses may depend on the specific signals from different cellular microenvironments.

Angiogenesis regulation involves multiple genes and pathways. We also examined the effects of DHI on expression of other angiogenesis factors. DHI did not significantly alter the expression of Tie-2, ANG1, ANG2, FGF-2, and PECAM in KKAy mice post HLI. It also did not significantly alter the expression of CXCL12, PECAM, ANG1, ANG2, Tie-2 and HIF1α, but did significantly upregulate the expression of FGF-2 and CXCR4 in STZ-induced diabetic mice. DHI had a trend, although statistically not significant in upregulation of VEGFR-1 expression. This seemingly inconsistent results may be because of the coexistence of pro- and anti-angiogenic substances in DHI that renders a diverse outcome as far as individual gene regulation is concerned.

Previous studies in animal models of ischemia [45,25] and limited human clinical trials [46] have documented that EPCs significantly augmented ischemia-induced angiogenesis. Furthermore, animal studies have shown that EPCs contribute to more rapid recovery of blood flow in the ischemic areas by augmenting vascular regeneration through formation of structural component of capillaries and secreting angiogenic growth factors [47,48]. Our studies revealed that DHI administration increased the number of EPCs in the peripheral blood circulation in genetic mice to the same level as approved for clinical use drug Plerixafor octahydrochloride (formerly known as AMD3100). The increase in the number of circulating peripheral blood EPCs was positively correlated with the ischemic limb recovery. Taken together, our findings suggested that DHI administration improved ischemia-induced angiogenesis *in vivo* through a VEGF-dependent mechanism and mobilization EPCs into peripheral blood could play a significant in this improved recovery process.

As a modern Chinese medicine, the chemical complexity of DHI has been well uncovered by recent studies [8–10]. Although we have yet to identify the specific component(s) in DHI that promote the growth of new vessels in order to supply sufficient blood flow and that ameliorate glucose metabolism, the complex formula of a Chinese medicine serves as a native drug combination and has the advantage to treat a disease from multiple target points and to regulate integrally, which can overcome the limitation of single therapy. Hydroxysafflor yellow A, salvianolic acid A/B and danshensu are among the main active chemical components in DHI. It has been confirmed that salvianolic acid A decreased blood glucose levels by improving glucose metabolism in STZ-induced type 2 diabetic rats. Liu M. et al. have reported that DHI reduced serum cholesterol and glucose levels in db/db mice [44]. In our study, we demonstrate that DHI also decreased blood glucose levels and improved the glucose tolerance in both genetic and chemically induced diabetic mice, but did not affect serum cholesterol levels (data not shown). This experimental difference may be due to the shorter delivery time and the use of different murine diabetic models (db/db vs. KKAy mice).

Hyperglycemia may be a major vascular risk factor in DM patients with PAD, therefore, a good glycemic control in all patients with PAD and diabetes may be essential in order to prevent diabetes-associated development and progression of microvascular complications. We hypothesized that improvement of diabetic peripheral arterial disease by DHI might be partly dependent on the control of glucose levels. To further elucidate the underlying mechanisms why DHI improved glucose homeostasis, we enriched the overlapping signaling pathways involved in angiogenesis and glucose tolerance using Ingenuity Pathway Analysis. Among them, PPARγ, PPARδ, CREBBP and PPARGC1A are shown to be highly associated with angiogenesis and glucose tolerance synchronously. The PPAR family consists of three ligand-activated nuclear receptors: PPARα, PPARδ and PPARγ. While PPARα is predominantly expressed in liver, heart, kidney, and brown adipose tissue and PPARγ is primarily expressed in adipose tissue, PPARδ is the most ubiquitously expressed. These PPARs have important roles in the regulation of glucose and fatty acid metabolism, cell differentiation and immune function. In recent years there has been a great interest in the actions of PPARα and PPARγ. PPARδ has roles in metabolism similar to PPARα and PPARγ such as improving glycemic control, decreasing insulin resistant and elevating high-density lipoprotein [49–51]. In addition, PPARδ was reported to have pro-angiogenic effects [5] unlike PPARα and PPARγ [52]. Results of our study showed that upregulation of PPARδ in both genetic and chemically-induced diabetic mice played an important role in glycemic control and angiogenesis, suggesting that DHI increased blood flow recovery and improved the glucose tolerance may be mediated through DHI-dependent upregulation of the PPARδ and activation of its downstream genes.

## Conclusion

In summary, our findings demonstrate that Chinese medicine DHI is efficacious for diabetic PAD in two mouse models by simultaneously promoting angiogenesis and enhancing EPC mobilization via a VEGF/VEGFR-2-dependent mechanism and improving glucose homeostasis via activation of PPARδ signaling. Further studies are needed to confirm and better understand the underlying mechanisms of DHI-mediated therapeutic effects, but these data may represent a supplementary non-invasive revascularization treatment method for PAD patients with diabetes.
